# The influence of social capital and socio-economic conditions on self-rated health among residents of an economically and health-deprived South African township

**DOI:** 10.1186/1475-9276-10-51

**Published:** 2011-11-15

**Authors:** Jane M Cramm, Anna P Nieboer

**Affiliations:** 1 Institute of Health Policy and Management, Erasmus University Rotterdam, P.O. Box 1738, 3000 DR Rotterdam, the Netherlands

**Keywords:** social capital, social determinants, socioeconomic status, health status, South Africa

## Abstract

**Background:**

Surprisingly few studies have investigated the interplay of multiple factors affecting self-rated health outcomes and the role of social capital on health in developing countries, a prerequisite to strengthening our understanding of the influence of social and economic conditions on health and the most effective aid. Our study aimed to identify social and economic conditions for health among residents of an economically and health-deprived community.

**Methods:**

Data were gathered through a survey administered to respondents from 1,020 households in Grahamstown a suburb in the Eastern Cape, South Africa (response rate 97.9%). We investigated the influence of social and economic conditions (education, employment, income, social capital, housing quality and neighborhood quality) on self-rated health. We used ordinal logistic regression analyses to identify the relationship of these conditions and self-rated health.

**Results:**

Our study found that education and social capital positively correlated with health; unemployment, poor educational level and advanced age negatively correlated. We found no significant correlations between self-rated health and housing quality, neighbourhood quality, income, gender, or marital status.

**Conclusion:**

We highlight the possible impacts of social capital, employment, and education on health, and suggest that health outcomes may be improved through interventions beyond the health system: creating job opportunities, strengthening social capital, bettering educational systems, and promoting educational access. Policymakers should consider the benefits of such programmes when addressing health outcomes in financially distressed districts.

## Background

People at the bottom of society are faced with the worst living conditions and report the worst health outcomes. Regardless the country these poor people live in, what type of health insurance they have or do not have, and the level of health care they receive, they still have the worst health of all [1]. These disparities cannot be explained by biological differences. The World Health Organization [2] holistically viewed the social determinants of health, concluding that global health and illness follow a social gradient; lower socioeconomic positions are consistently correlated with poorer health. These avoidable health inequalities arise because of the circumstances in which people are born, live, work, and age, including the adequacy of health care systems. The conditions in which people live and die are, in turn, shaped by social and economic forces. Together, the structural determinants and conditions of daily life constitute the socioeconomic determinants of health [3]. Research has shown that, despite the potential of the health system in developing countries in reducing socioeconomic inequality, such inequality is related to factors beyond the scope of health authorities and the health care delivery system [4,5]. Therefore, health systems will not be able to achieve equity without commitments and interventions beyond the health sector [6-10].

Overcoming health inequity requires evidence based on measurement of the social determinants of health [11]. The act of measurement itself is a challenge as the poor are often missing from official statistics [12]. To strengthen our understanding of health inequity, the social determinants of health, and the most effective means of improvement, studies must be conducted in economically and health-deprived communities [13-15].

Evidence suggests that individual socioeconomic resources (education, employment and income) affect health [15-20]. In addition, individual level experiences of environmental conditions such as housing quality (e.g. leaking roof) and neighborhood quality may affect individual health [16-20]. Neighbourhood crime is an example of neighbourhood quality representing residents' perceived threat to personal safety and environmental stress [20]. A growing body of evidence has demonstrated that higher social capital is associated with improved health conditions [21-25]. Putnam referred to social capital as, 'features of social organization such as networks, norms, and social trust that facilitate coordination and cooperation for mutual benefit' [26]. While social capital is recognised to have an important influence on health, the specific mechanisms of this association remain incompletely defined. Researchers have suggested three possible mechanisms to explain how social capital produces health benefits: (1) influence health-related behaviors, (2) influence access to services and amenities and (3) affect psychosocial processes [26,27]. Rogers' diffusion of innovations theory [28] suggests that social capital may promote the diffusion of health-related knowledge and interventions within communities and neighbourhoods. Because social capital reflects the social processes, norms, and trust among community members, it is an important resource for community health promotion. Social capital can provide valuable, up-to-date, and timely health information and promotes the diffusion of health-related interventions [27]. The success of community interventions often lies in their ability to engage and strengthen social capital. They are grounded in the notion that healthy behaviour is better shaped by influencing social norms and negotiating collective identities, rather than providing individuals with factual information. This theory is supported by research demonstrating that health behavior is influenced more by perceptions than knowledge [29-32]. Individuals obtain health information primarily through social connections [33], and communities with high levels of social capital are more effectively in exercising social control over health behaviours [27]. Poortinga [34] demonstrated that social capital is an important determinant of health and healthy behaviour, and suggested that healthy behaviours mediate the relationship between social capital and health. In addition, social capital may affect psychological processes, including the provision of affective support and mutual respect [35], that may discourage deviant health behaviours [34]. Furthermore, it may shelter people from the harmful effects of unemployment and poverty [35,36], or provide a buffer against the adverse effects of stress and poverty [37].

### Study aims

Many studies investigated the relationship between socio-economic conditions and health outcomes. Few studies, however, investigated the role of social capital as a socio-economic condition on health. Although there are some studies that have tested the relationship between social capital and health, they all have one major limitation: poor people or those within deviant or marginalized communities have been excluded [38]. Our study aimed to identify the role of social capital in addition to other well-known socio-economic conditions (education, employment, income, housing quality and neighborhood quality) on health for residents of an economically and health-deprived community in South Africa. We hypothesize that social capital affects health among poor communities.

## Methods

### Participants and sampling

South Africa's historical pattern of racially segregated spatial planning may have led to poor health and created different health patterns. Rhini is characterised by high levels of poverty and unemployment and low educational levels [39,40]. The survey forming the foundation of our study covered the informal residential areas within the Grahamstown municipal district, referred to as Grahamstown East, or Rhini. A systematic sampling technique was employed to sample the 11,127 households in the 2,500 square kilometres of Rhini [41]. Public structures such as civic offices, schools, and churches were omitted. All neighbourhoods in the sample were proportionately represented in the study population. Sampling began from a randomly chosen point and moved systematically through each of the 20 neighbourhoods. Every tenth household was selected for inclusion in the sample, resulting in 1,042 targeted households. This method ensured that all households in all neighbourhoods of Rhini stood an equal chance of inclusion.

Respondents were then identified in each targeted household. Eligibility requirements were age (at least 18) and length of residency (at least 6 months of the past year). One respondent from each targeted household was selected using a Kish grid to ensure that all eligible persons in the household stood an equal chance of being included [42]. Up to four attempts were made to interview the selected respondents, with success in 1,020 (97.9%) of the 1,042 households. In the remaining cases, respondents were absent for all four visits, impaired by age or health, or disinterested/unwilling.

### Survey instrument

Professional interviewers from Development Research Africa, an organisation with experience in national probability-based sampling in deep rural and urban areas, administered the questionnaires in 2007. Most items were closed-ended, with a supplied set of response options. The interviewers gathered demographic information about the participants, including gender, age, health status, education level, living arrangements, and employment.

### Measurements

Health status, however measured, is more than just the absence of disease. It includes a set of factors that relate to how individuals feel as well as function in society and the environment [43]. We determined self-reported health status with the question, ''How would you describe your overall state of health these days? Would you say it is (4) excellent, (3) very good, (2) good, (1) fair, or (0) poor?'' thus defining it as the individual's personal evaluation of their overall health without referring to any one component of physical, social, mental, or functional health. Public health research has typically used this measure as a proxy for ''objective'' but difficult to measure health outcomes. The test-retest reliability of self-rated health has been shown to be high across subgroups of age and sex [44].

To investigate socio-economic conditions affecting health, the questionnaire established employment, household income (ranging from (1) between 0 and 100 South African Rand to (13) more than 7,000 South African Rand), age, gender, marital status, and education, the last having the answers: "no education", "some primary and/or secondary education", "matric and higher education".

Following Putnam, we measured social capital through respondents' perceptions of the norms of reciprocity and their trust in others, factors that facilitate cooperation for mutual benefit [26]. Social capital was assessed with three statements for which respondents rated their level of agreement on a four-point scale: "People in this neighbourhood are friendly", "people in this neighbourhood help each other without having to be asked", and "people in this neighbourhood trust their neighbours". Social capital scores are derived by summing the response choices on these three items and dividing it by the corresponding number of items. The Cronbach's alpha of the social capital instrument was 0.87, indicating reliability.

We assessed housing quality with a dichotomised item, "Has the roof leaked in the past year?' and by asking respondents to identify whether their house is built formally (brick/cement block) or informally (shack/traditional pole/mud house), to which they could answer 'yes', or 'no'.

Neighbourhood quality was estimated with two survey items: "Residents in this neighbourhood get fair values for their rental rates" and "There is not a lot of crime in this neighbourhood". Respondents rated their level of agreement to both neighbourhood quality survey items on a four-point scale.

### Data analysis

We generated descriptive summary statistics and performed regression analyses to identify social and economic conditions (education, employment, income, social capital, housing quality and neighborhood quality) for residents of an economically and health-deprived community in South Africa. We applied an ordinal logistic regression model to account for the ordinal structure of the outcome variable. We defined the level of significance at *P *≤ 0.05; we additionally report results with a larger level of confidence (*P *≤ 0.001). The regression equation specification error test will be used to detect if our model is misspecified. Statistical Package for the Social Sciences (SPSS) version 17.0 software was used for all statistical analyses.

## Results

Respondents were mostly female (73%), had a median age of 38 (range: 18-98), and in one-third (33%) of the cases, were married. Thirty-five percent had no formal schooling or only primary education, 40% some secondary education, and 25% had matriculated or had higher education. One-half (50%) of respondents had had a roof leak in the past year; 72% lived in a formal house and 28% in an informal house (shack or mud dwelling). Table 1 provides descriptive statistics for the variables used in the ordinal logistic regression analyses.

**Table 1:** Descriptive statistics (*n *= 1,020; Grahamstown 2007).

Model	N	%	Mean	SD	Min	Max
**Health**			2.55	1.01	0.00	4.00
0 (poor)	17	2%				
1 (fair)	156	17%				
2 (good)	214	23%				
3 (very good)	405	43%				
4 (excellent)	143	15%				
***Socio-economic conditions/resources***						
Married	304	33%				
Male	249	27%				
Unemployment	579	62%				
Education						
None or some primary	325	35%				
Some secondary	375	40%				
Matric or higher	235	25%				
Income			1,250	2.13	0.00	> 7,001
Social capital			2.97	0.54	1.00	4.00
***Housing quality ***						
Leaky roof	471	50%				
Formally build house	670	62%				
***Neighbourhood quality***						
Crime in the area			2.03	0.89	1.00	4.00
Residents get value for their rental rates			2.19	0.74	1.00	4.00

Table 2 presents the results of the ordinal logistic regression analyses on self-rated health. These analyses revealed that unemployment, poor educational level and increasing age significantly contributed to poor health status within this community, while social capital significantly promoted good health. No significant correlations were found between self-rated health and housing type, leaky roof, neighbourhood quality, income, gender, or marital status (table 2). The OR for employment was 2.058, indicating that the ratio of the odds for good health increased by 2.058-fold for the employed compared to the unemployed, assuming all other factors in the model remained constant. Having non or only some primary education decreased the odds of good health by factor 0.464 compared to those with higher educational levels (matric or higher). Similarly, an increase in age was related to a decrease in the odds for good health by a factor 0.942. Higher levels of social capital was related to an increased probability of good health, as indicated by a 1.650 OR, meaning that the odds for good health increased by factor 1.650 for community members with higher levels of social capital, assuming all other factors in the model remained constant. This model explained 35.3% of the variance in the outcome measure (as indicated by Nagelkerke's R^2^). The regression equation specification error test showed that our regressions model is not misspecified.

**Table 2:** Ordinal logistic regression analyses (*n *= 1,020; Grahamstown 2007).

Model	B	SE	Wald Z	P	OR
Intercept 1 ≤ 0	-6.061	0.589	105.177	≤ 0.001	0.002
Intercept 2 ≤ 1	-3.253	0.532	37.413	≤ 0.001	0.039
Intercept 3 ≤ 2 Intercept 4 ≤ 3	-1.700 0.987	0.522 0.522	10.610 3.502	≤ 0.001 0.061	0.183 2.683
***Socio-economic conditions/resources***					
Married	0.029	0.135	0.045	0.833	1.029
Male	-0.156	0.142	1.204	0.273	0.856
Age	-0.059	0.005	139.069	**≤ 0.001**	0.942
Employment	0.722	0.154	22.094	**≤ 0.001**	2.058
Education (none or some primary)	-0.767	0.194	15.617	**≤ 0.001**	0.464
Education (some secondary)	-0.085	0.165	0.269	0.604	0.919
Income	0.001	0.035	0.000	0.985	1.001
Social capital	0.501	0.119	17.845	**≤ 0.001**	1.650
***Housing quality***					
Leaky roof	-0.050	0.134	0.138	0.710	
Formally build house	-0.029	0.150	0.037	0.847	0.971
***Neighbourhood quality***					
Crime in the area	0.080	0.072	1.255	0.263	1.083
Residents get value for their rental rates	-0.156	0.088	3.186	0.074	0.856
*Test*					
Overall model evaluation Wald test		*Chi^2^* 374.550	*df* 12	*P* ≤ 0.001	
R^2 ^Nagelkerke	35.3%				

Notes: reference group of the ordinal logistic model is excellent health (4). B = unstandardized regression coefficient.

## Discussion

Our study aimed to identify the social and economic condition affecting health among residents of Rhini, an economically and health-deprived community in the Eastern Cape of South Africa. We hypothesized that in addition to well-known socio-economic conditions, social capital affects health among poor communities. As expected, our study showed that social capital affected health in poor communities. Our study corroborates the importance of social capital. Social capital may have promoted the diffusion of health-related knowledge, since people obtain much of their health information through social connections [33]. Higher levels of social capital may therefore have led to increased knowledge, as well as healthy and disease-preventive behaviours. These results agree with Poortinga [34] identifying that social capital is an important determinant of health and healthy behaviour, and suggested that healthy behaviours mediate the relationship between social capital and health. Lack of social capital may lead to increased feelings of loneliness and stress, which negatively affect health outcomes and lead unhealthy behaviours such as drinking and smoking. Furthermore, social capital can affect psychological processes like support and mutual respect, which may discourage deviant health behaviours [34], improve awareness of health and disease-related issues [27,28] and improve overall well-being outcomes in poor communities [37].

Interventions aimed at strengthening social capital may thus reduce health inequalities. Pronyk and colleagues [5] found in their longitudinal study that social capital could be intentionally generated in relatively short programmatic time frames. They conducted an intervention in rural South Africa that combined group-based microfinance with participatory health education; within two years they had simultaneously promoted health, social capital, and economic development. This is in contrast to Putnam's proposal that the accumulation of social capital takes place only very slowly [26]. Public health practitioners and policy makers have recently turned to more comprehensive and participatory approaches to enhancing social capital, and thereby improving health outcomes [45]. Instead of adopting a top-down approach, policy makers increasingly work with community members to plan and implement health programmes. Farquhar and colleagues [46] found an association between health and social capital, and determined that increased social capital generated through community-based participatory interventions led to significantly improved health outcomes. Our findings also highlighted this effect of social capital.

This study revealed that unemployment, poor educational level and advanced age significantly contributed to poor health. With this article we wish to highlight the impacts of employment and education on health, and to suggest that health outcomes may be improved with interventions aimed at creating job opportunities, strengthening educational systems and promoting universal educational access. Health may be improved with interventions beyond the health sector such as group-lending microfinance schemes that could create employment and strengthen social capital at the same time [5,19]. Social capital could be further strengthened by creating meeting opportunities for neighbourhood residents [47]. Such policies may also improve a wide range of health issues by promoting healthy behaviour, disease awareness, and disease-preventive behaviour. In addition, health may be improved by strengthening educational systems and promoting universal educational access.

Some limitations must be considered when interpreting our study findings. First, the cross-sectional nature of the data limited our ability to draw causal inferences or determine the directionality of associations. Our establishment of significant associations is, however, an important step for further studies investigating directionality. Second, while Uphoff [48] distinguished between structural and cognitive dimensions of social capital, we only measured cognitive social capital. Cognitive social capital derives from individuals' perceptions of social capital resulting in norms, values and beliefs that contribute to cooperation. Structural social capital deals with individuals' actual behaviours and mainly takes the form of networks and associations.

## Conclusion

Our study demonstrated that social capital, employment and education are significantly related to health in the low-income South African township of Rhini. These findings are important in understanding the struggles of the lowest socio-economic stratum. While existing health policies in South Africa emphasise disease treatment, our findings suggest that a greater emphasis on social environment, employment, and education could be more beneficial. We especially urge the designers of health-related interventions to consider the possible effects of social capital on health outcomes. We trust that these findings will be useful for policymakers, governments, municipalities, and organisations faced with the task of promoting good health.

## Competing interests

The authors declare that they have no competing interests.

## Authors' contributions

AN participated in the design of the study and JC drafted the manuscript. JC and AN performed the statistical analysis. Both authors have read and approved the final version of the manuscript.
